# Systematic analysis of mutation distribution in three dimensional protein structures identifies cancer driver genes

**DOI:** 10.1038/srep26483

**Published:** 2016-05-26

**Authors:** Akihiro Fujimoto, Yukinori Okada, Keith A. Boroevich, Tatsuhiko Tsunoda, Hiroaki Taniguchi, Hidewaki Nakagawa

**Affiliations:** 1 Laboratory for Genome Sequencing Analysis, RIKEN Center for Integrative Medical Sciences, Japan; 2 Department of Drug Discovery Medicine, Graduate School of Medicine, Kyoto University, Japan; 3 Laboratory for Statistical Analysis, RIKEN Center for Integrative Medical Sciences, Japan; 4 Department of Human Genetics and Disease Diversity, Graduate School of Medical and Dental Sciences, Tokyo Medical and Dental University, Japan; 5 Laboratory for Medical Science Mathematics, RIKEN Center for Integrative Medical Sciences, Japan

## Abstract

Protein tertiary structure determines molecular function, interaction, and stability of the protein, therefore distribution of mutation in the tertiary structure can facilitate the identification of new driver genes in cancer. To analyze mutation distribution in protein tertiary structures, we applied a novel three dimensional permutation test to the mutation positions. We analyzed somatic mutation datasets of 21 types of cancers obtained from exome sequencing conducted by the TCGA project. Of the 3,622 genes that had ≥3 mutations in the regions with tertiary structure data, 106 genes showed significant skew in mutation distribution. Known tumor suppressors and oncogenes were significantly enriched in these identified cancer gene sets. Physical distances between mutations in known oncogenes were significantly smaller than those of tumor suppressors. Twenty-three genes were detected in multiple cancers. Candidate genes with significant skew of the 3D mutation distribution included kinases (*MAPK1*, *EPHA5*, *ERBB3*, and *ERBB4*), an apoptosis related gene (*APP*), an RNA splicing factor (*SF1*), a miRNA processing factor (*DICER1*), an E3 ubiquitin ligase (*CUL1*) and transcription factors (*KLF5* and *EEF1B2*). Our study suggests that systematic analysis of mutation distribution in the tertiary protein structure can help identify cancer driver genes.

Identification of driver genes from the mutation catalog is one of the most important issues in cancer genetics research. The identified driver genes can lead to development of new drugs and elucidate mechanisms of carcinogenesis^1^,^2^. One of the strongest signatures of cancer driver genes is the frequency of mutated samples and/or number of mutations. In recent studies, statistical comparison between the number of observed and expected mutations, i.e. gene burden test, has been performed and many driver genes have been discovered^3^,^4^. Although this framework has been used to successfully identify new driver genes and pathways, recent studies reveal that most cancers are very heterogeneous, and many low-frequency driver genes exist in the long-tailed mutated gene lists^3^,^4^. To discover low-frequency driver genes from huge lists of mutations, application of a new algorithms that focuses on new aspects of driver genes is required.

Driver genes are generally classified into oncogenes and tumor suppressor genes (TSGs). Mutations in oncogenes render the gene constitutively active (gain of function) or confer a different function (change of function)^2^. On the other hand, mutations in TSGs reduce activity of the gene^2^. Since the amino acid residues that can activate protein function tend to be limited, mutations in oncogenes are likely to be tightly clustered to a few functionally important codons^2^. In the TSGs, missense mutations would be distributed in functionally important domains, such as the DNA binding domain in the *TP53* gene^5^. This expectation predicts that mutations in driver genes are not randomly distributed, and analysis of mutation position in a gene can identify driver genes^6^,^7^,^8^.

In this study, we examined the skew of mutation distributions in the tertiary structures of the proteins. Our analysis revealed a different pattern of mutation distribution between oncogenes and TSGs, and identified driver genes and pathways. We consider that our method can complement traditional gene burden tests and contribute to a better interpretation of mutations in cancer genomes.

## Results

### Analysis method

**Figure 1:**
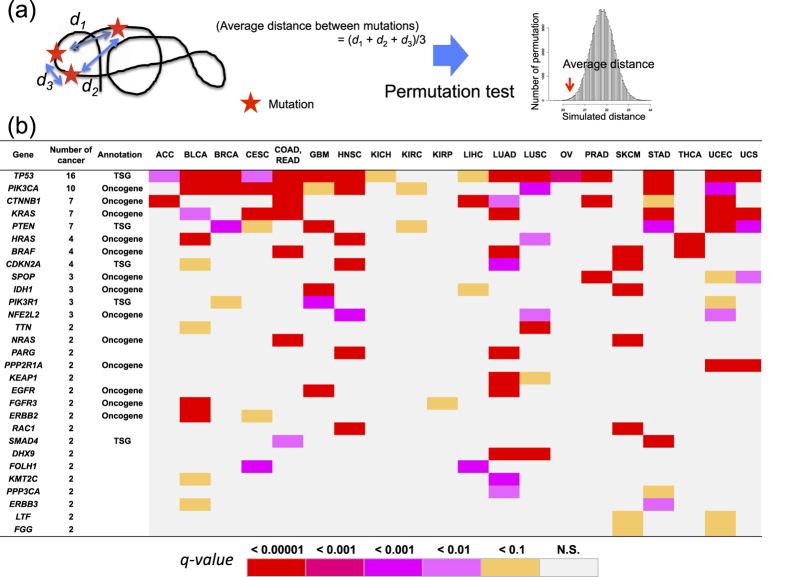
Explanation of the 3D permutation test and significant genes. (**a**) 3D permutation test procedure. Average distance between mutations in the 3D structure is calculated. Significance was tested by a permutation test. (**b**) Significant genes in multiple cancers. Twenty-three genes were significant in multiple cancers. Annotation was based on Vogelstein *et al*.^24^. Two genes (*CHEK2* and *NR1H2*) were identified in multiple cancers due to single hotspot in each gene. However, mutant alleles of these two hotspots were found in the dbSNP database (rs146546850 of *CHEK2* and rs55817866 of *NR1H2*) and population allele frequencies were not low (0.7% for rs146546850 and 9.7% for rs55817866), therefore we removed these genes from this table.

In the present study, we developed a novel method to analyze mutation position in the tertiary (3 dimensional [3D]) structure of proteins. Since biochemical activities of proteins depend on their structure *in vivo*, it is reasonable to analyze mutation positions in the context of their 3D structure. To evaluate 3 dimensional accumulation of mutations, we applied a permutation test (3D permutation test) (Fig. 1a and Supplementary Fig. 1). Protein 3D structures were downloaded from the Protein Data Bank (PDB), and the amino acid sequence of the protein structure was aligned to that of the reference genome using MAFFT^9^. Based on this alignment, mutations in the reference genome were mapped onto the 3D structure. For each gene with *n* mutations in the sequence covered by the 3D structure, the average pairwise Euclidean distance between the *n* mutations (observed average distance) was calculated.

To obtain the null distribution, *n* positions were randomly selected from the amino acid residues, and the average distance (simulated average distance) was calculated. This step was repeated 10^4^~10^6^ times to generate the null distribution of the simulated average distances. The significance of the observed distance was obtained from the null distribution (Fig. 1a, and Supplementary Fig. 1). Thus, the skew of mutation position was evaluated by the *p-value*. In addition to the 3D permutation test, we performed a simple permutation test for the observed average distance on the protein primary structure (1D permutation test).

### Analysis of cancer somatic mutation data

We applied this method to exome sequence data from 7,215 samples of 21 types of cancer released from the TCGA project (Supplementary Table 1)^10^,^11^,^12^,^13^,^14^,^15^,^16^,^17^,^18^,^19^,^20^,^21^,^22^,^23^. To remove possible germline SNVs, we examined two independent filters: Filter 1, removal of somatic mutation candidates that were present in the 1000genome common SNVs set, and Filter 2, removal of somatic mutation candidates were found in dbSNP with a validation status of “YES”. Filter 2 was more conservative but removed known cancer hotspots, therefore we adopted the result from filter 1 (Results from the filter 2 and comparison of the two filters can be found in Supplementary Tables 2 and 3 and the Supplementary information).

**Figure 2:**
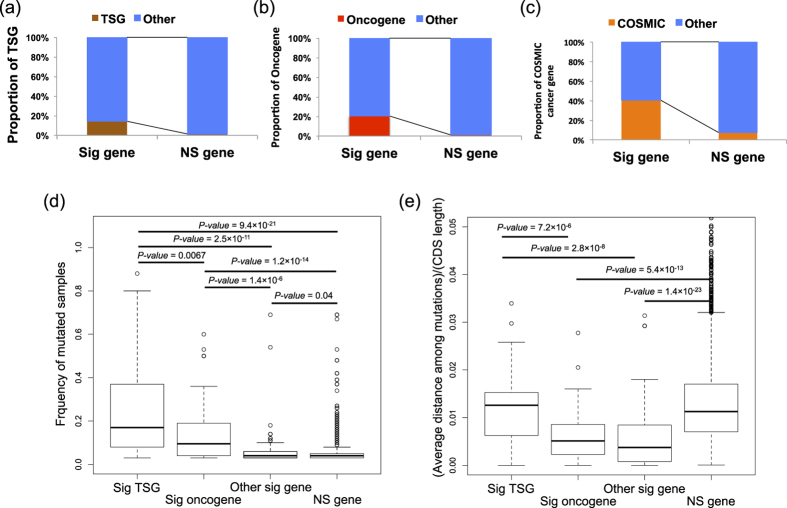
Analysis of candidate driver genes. **(a)** Proportion of oncogenes. Oncogenes were enriched in the significant gene set (Fisher’s exact test; *p-value* = 7.4 × 10^−18^; odds ratio = 29.5). Sig gene - significant gene. NS gene - not significant gene. **(b)** Proportion of TSGs. TSGs were enriched in the significant gene set (Fisher’s exact test; *p-value* = 7.1 × 10^−10^; odds ratio = 11.8). **(c)** Proportion of genes in COSMIC cancer gene census. COSMIC cancer genes were enriched in the significant gene set (Fisher’s exact test; *p-value* = 5.9 × 10^−19^; odds ratio = 9.0). **(d)** Frequency of mutated samples for significant oncogenes, significant TSGs, the other significant genes and non-significant genes. Frequency of mutated samples of TSGs was significantly higher than that of oncogenes, the other significant genes and not significant genes. *P-values* were obtained by Wilcoxon’s rank sum test. Unadjusted *p-values* were shown. **(e)** Adjusted average distance between mutations in significant oncogenes, significant TSGs, the other significant genes and not significant genes. Average distance between mutations was adjusted by length of the coding region. Adjusted average distance of TSGs was significantly larger than that of oncogenes, and the other significant genes. Adjusted average distance of oncogenes, the other significant genes was significantly smaller than that of not significant genes. *P-values* were obtained by Wilcoxon’s rank sum test. Unadjusted *p-values* were shown.

Using the filtered SNV set, we analyzed 3,622 genes with ≥3 missense mutations in the 3D structure using the 3D permutation test. AML did not have a sufficient number of somatic mutations, and no genes passed the analysis criteria. Analysis with different minimum missense mutation cutoffs (≥4, 5, and 6) is provided in the Supplementary information. Quantile-quantile (Q-Q) plots of the permutation test did not show a systematic inflation of test statistics (Supplementary Fig. 2). After adjustment for multiple testing on the number of the analyzed genes, 188 genes (106 unique genes) had mutations in the >3% of the samples with a false discovery rate (FDR) *q-value* ≤0.1 (Supplementary Table 2). Of the 21 evaluated cancer types, significant genes were identified in 20 cancer types. Of the 106 significant genes, 31 were significant across multiple cancers (Fig. 1b). To examine the reliability of the result, we examined whether known cancer genes were identified by the 3D permutation method. We adopted the annotation of tumor suppressor genes (TSGs) or oncogenes as defined by Vogelstein *et al.*^24^, and CSOMIC cancer gene census. The set of significant genes that were mutated in more than 3% of the samples were enriched for tumor suppressor genes (TSGs), oncogenes and COSMIC cancer genes (TSG; Fisher’s exact test *p-value* = 7.1 × 10^−10^, and odds ratio = 11.8, oncogene; *p-value* = 7.4 × 10^−18^, and odds ratio = 29.5, and CSOMIC cancer gene census gene; *p-value* = 5.9 × 10^−19^, and odds ratio = 9.0) (Fig. 2a–c), suggesting that our analysis can successfully identify driver genes.

We then analyzed frequency of mutated samples and observed average distance between mutations among significant oncogenes, TSGs, other significant genes and non-significant genes. Known TSGs were found to be mutated in a significantly larger number of samples than oncogenes (Fig. 2d). Known TSGs and oncogenes were mutated in a significantly larger number of samples than the other genes (Fig. 2d). Since most of the TSGs and oncogenes were previously detected by frequency of mutated samples, it is reasonable that their frequencies are higher than the other genes. When conditioned on the length of coding sequences (CDS) of the respective genes, the adjusted average distances of the oncogenes were significantly lower than those of the TSGs (*p-value* = 7.2 × 10^−6^), indicating that the mutations in the oncogenes were more tightly clustered than those of the TSGs (Fig. 2e). The adjusted average distances of the other significant genes were significantly smaller than those of the TSGs (*p-value* = 2.8 × 10^−8^), but not more than those of the oncogenes. These results suggest that the other significant gene set likely includes potential oncogenes, which were difficult to identify using conventional frequency based methods.

### Comparison with other methods

We compared our method with the gene burden test and test for mutation clustering. We applied the 3D permutation method to the mutation lists of BRCA, KIRC, LUSC and UCEC, which were analyzed by Lawrence *et al.*^22^, and compared the results for the testable genes by the 3D permutation method (the number of mutations in the 3D structure ≥3) (Supplementary Table 4, and Supplementary Fig. 3). First, we compared our result with that from MutSigCV, which is a gene burden test (Supplementary Fig. 3a). In the four cancer types, 16 genes were significant by both methods. Nineteen genes were found only by MutSigCV, likely due to the wide distribution of mutations in these genes. Fifty-nine genes were uniquely identified by our method. Second, we compared the 3D permutation method with results for LUSC and UCEC by MutSigCL^22^ and OncodriveCLUST^6^, which are the methods to detect mutation clusters in the primary structure. In the four cancer types, sixty-eight genes were significant by only one method, and 19 and 9 genes were detected by two and three methods, respectively (Supplementary Fig. 3b). Thirty-five genes were identified as significant only by the 3D permutation method, which included the *FGFR3*, *HRAS*, and *KEAP1* genes. Third, we compared the 3D permutation method with combined MutSig result, which were generated by merging *p-values* of three methods^22^ (Supplementary Fig. 3c). In the four cancer types, 27 genes were significant by the both methods. Twenty-two genes were found only by MutSig. Fifty-one genes were uniquely identified by our method, which included the *FGFR2*, *HRAS, NFE2L2* and *DICER1* genes These results suggest that the 3D permutation method can compliment the gene burden test and the previous methods to detect mutation clusters, and identify new candidates, although analysis is restricted to genes with 3D structures available.

### Genes with skew in mutation distribution

**Figure 3:**
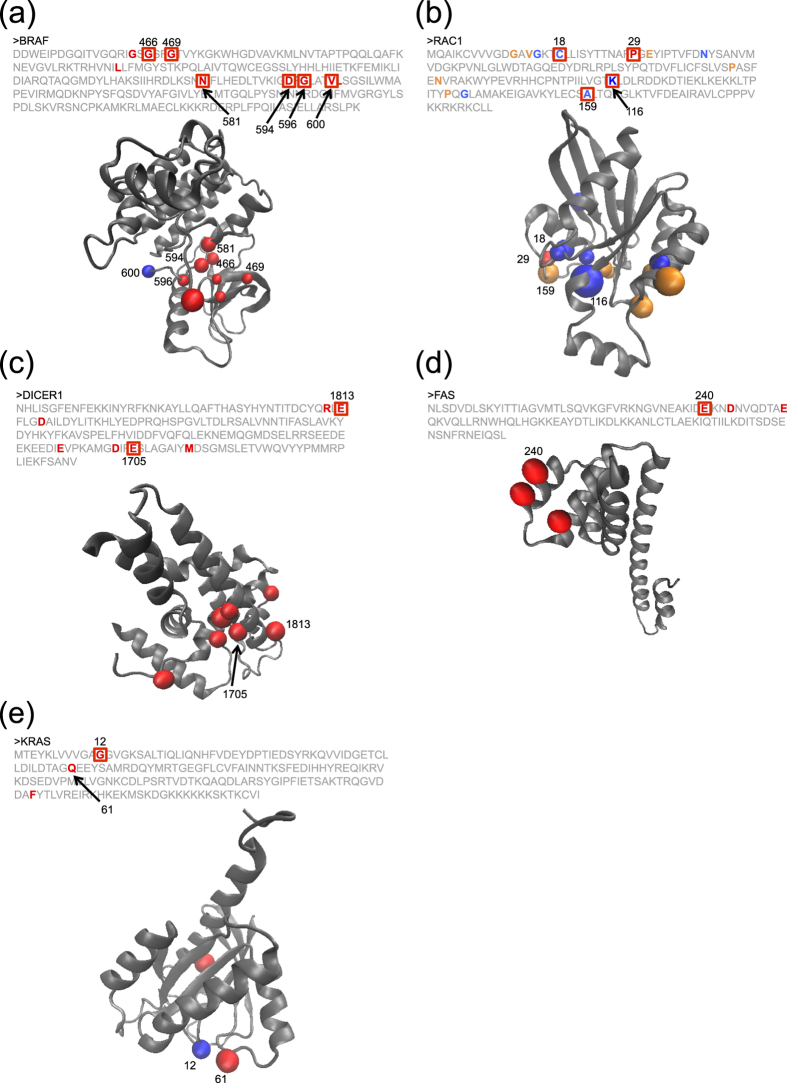
Examples of the significant genes identified by the 3D permutation. Primary structure, 3D structure, and mutations are shown. Amino acid sequence and 3D structure are shown. Recurrently mutated codons are indicated by the square in the amino acid sequence, and the position of the codons are shown in the 3D structures. Figures of the 3D structure were generated by VMD software. **(a)** *BRAF*. *q-value* of 3D permutation (*q-value*_3D_) <10^−6^. *q-value* of 1D permutation (*q-value*_1D_) <10^−6^. Codon 600 (hotsopt) is shown in blue in the 3D structure. **(b)** *RAC1*. NHSC: *q-value*_3D_ <10^−6^. *q-value*_1D_  =  n.s. (not significant). SKCM: *q-value*_3D_ <10^−6^. *q-value*_1D_ = 0.0067. Mutations in NHSC and SKCM are shown in blue and orange, respectively. The common mutation is shown in red. **(c)** *DICER1*. UCEC: *q-value*_3D_ = 0.0044. *q-value*_1D_ = n.s. Sequence in the structure correspond to RNase III domain in *DICER1*. **(d)** *FAS.* CESC; *q-value*_3D_ = 0.025. *q-value*_1D_ = n.s. Change at codon 240 causes loss of interaction with FADD^38^. Two other mutations are close to the codon 240 in the 3D structure, suggesting that these positions are functionally important as well. **(e)** *KRAS*. BLCA: *q-value*_3D_ = 0.0020. *q-value*_1D_ = n.s. Codon 12 (hotsopt) is shown in blue in the 3D structure.

The genes, *TP53, PIK3CA, CTNNB1, KRAS, PTEN, HRAS, BRAF, CDKN2A, PIK3R1, NFE2L2, SPOP*, and *IDH1* showed a significant skew in the observed mutation distribution in 3 or more different types of cancers. Most of them are known TSGs or oncogenes, suggesting they have universal roles in carcinogenesis. While the mutation distribution for most of genes showed similar patterns, *CDKN2A* gene in the HNSC samples had an additional hotspot (Supplementary Fig. 4). Additionally, we obtained important observations of *BRAF*. In LUAD, the distribution of mutation in BRAF was significantly skewed even after the removal of the known hotspot (codon 600) (Fig. 3a), suggesting functional effects of the non-hotspot mutations.

A significant skew was observed for eighteen genes in exactly 2 different types of cancers and 6 of them (*NRAS, PPP2R1A, EGFR, FGFR3, ERBB2* and *SMAD4*) are classified as either a TSG or an oncogene. Additionally, *RAC1* and *KEAP1* are known to be important driver genes^25^,^26^ (Fig. 3b). *KMT2C*, also called *MLL3*, encodes a chromatin regulator^27^. *DHX9* and *PARG* had the same mutational hotspot across cancer groups (Supplementary Table 2). *PARG*, a poly (ADP-ribose) glycohydrolase, is known to remove ADP-ribosylation of some DNA repair proteins recruited to DNA damage sites and can influence the activity of the DNA repair system along with PARP1^28^. *DHX9* encodes a DEAH-box DNA/RNA helicase that catalyzes the ATP-dependent unwinding of double-stranded RNA and DNA-RNA complexes^29^,^30^. DHX9 plays important roles in multiple cellular pathways, including protein translation, RNA splicing, maintenance of genomic stability and apoptosis^29^,^30^. Since *DHX9* and *PARG* were mutated in less than 5% of the samples, they would have been difficult to identify through conventional frequency based methods. In addition, known cancer driver genes that are often mutated at low frequency among samples, such as *BRAF* in LUAD, *NFE2L2* in HNSC, *CDKN2A* in LUAD and *PIK3CA* in GBM, were identified by the 3D permutation test. This result strongly suggests the important role of these genes in certain cancers.

Seventy-five genes were identified in only one type of cancer. Of these, 58 genes were neither classified as an oncogene nor a TSG, and 54 were not in the COSMIC cancer gene census. The seventy-five genes included TSGs (*VHL*, *BAP1*, *HNF1A*, *PBRM1*, and, *RB1*), oncogenes (*AKT1*, *FGFR2*, *GNAS*, *MAP2K1*, and *MET*), kinases (*MAPK1*, *EPHA5*, *ERBB3*, and *ERBB4*), an apoptosis related gene (*APP*), an RNA splicing factor (*SF1*), a miRNA processing factor (*DICER1*), an E3 ubiquitin ligase (*CUL1*) and transcription factors (*KLF5* and *EEF1B2*) (Supplementary Table 2) (*DICER1* and *FAS* are shown as examples in Fig. 3c,d).

### Pathway analysis

To identify pathways related to the putative driver genes, we analyzed pathway enrichment in the reactome database, and identified 326 pathways with an FDR <0.1 (Supplementary Table 5). Since most of these pathways were already known critical pathways, we excluded genes annotated as oncogenes and TSGs, and carried out the same analysis for the remaining 71 genes. One hundred seventy-two pathways were significantly enriched (Supplementary Table 6), including pathways related to “axon guidance” and “semaphorin”. Genes related to the axon guidance pathway were identified in pancreatic and liver cancers^31^,^32^. Semaphorin is involved in tumor growth, invasiveness and metastasis, and our analysis provides supportive evidence of the importance of the semaphorin pathway in carcingenesis^33^.

### Comparison between the 3D and 1D permutation tests

We then compared the results between the 3D and 1D permutation tests (Note that 1D permutation was done for entire coding regions, but 3D permutation analysis was limited to coding region in the 3D protein structure) (Supplementary Fig. 5). For the 1D permutation tests, 141 genes (80 unique genes) had a significantly skewed observed average distance between mutations, 44 of which were commonly identified by both the 3D and 1D permutation tests (Supplementary Fig. 6). The number of significant genes found in the 3D analysis was larger than that of 1D analysis. We compared the proportion of known TSGs, oncogenes and COSMIC cancer genes among the genes identified by the both methods, only the 1D test, and only the 3D test. As expected, commonly identified genes had the highest proportion of these cancer-related genes (Supplementary Fig. 6). Genes identified by only the 3D test had a larger proportion of oncogenes, TSGs and COSMIC cancer genes than these of the 1D test (COSMIC cancer genes; *P-value* = 0.028, oncogenes and TSGs were not significantly different) (Supplementary Fig. 6).

Focusing on each gene, driver genes, such as *ERBB2, KIT, HNF1A* and *GNAS*, were not identified in the 1D analysis (Supplementary Fig. 7a–d). In addition to the known driver genes, our 3D analysis identified *CUL1* as a candidate driver gene (Supplementary Fig. 7e). CUL1, a known cell cycle regulator, is a member of the Skp1-Cul1-F-box ubiquitin ligase complex, which functions as both a tumor suppressor and oncogene through degradation of its target proteins^34^,^35^. Accordingly, 3D permutation analysis should be favored over 1D analysis as it confers several advantages including the efficient identification of oncogenes and TSGs.

We manually reviewed the genes identified only by the 3D permutation test, and found interesting examples. Several recurrently mutated codons, whose frequency was not enough high to be identified by the 1D analysis, were clustered in the 3D structure in *RAC1* of HNSC, *DICER1* of UCEC and *KIT* of SKCM (Fig. 3b,c and Supplementary Fig. 7b). One recurrently mutated codon and positionally close mutation(s) were found in *KRAS* of BLCA, *ERBB2* of CESC and *GNAS* of SKCM (Recurrently mutated codons were codon 12 of *KRAS*, codon 279 of *ERBB2* and codon 844 of *GNAS*. Codon 12 of *KRAS*, and codon 844 of *GNAS* are known as the hotspots of the genes) (Fig. 3e and Supplementary Fig. 7a,e). In *KRAS*, one mutation (codon 61) was close to the known hotspot (codon 12) in the 3D structure (Fig. 3e). Mutations at codon 61 were also observed in other cancers (COAD, READ, STAD and UCEC), and the mutation was known to have a functional effect^36^. These examples show that the analysis of mutation distribution in the 3D structure merge mutated codons, which would not be detected as a mutation cluster by 1D analysis, and thus increases detection power for driver gene identification.

## Discussion

The 3D permutation method successfully identified many known oncogenes, TSGs and promising candidates of cancer driver genes. Interestingly, our analysis identified a number of TSGs, in addition to the oncogenes, suggesting that skew of mutation distribution is a general feature of TSGs. Although the mutations in the oncogenes are tightly clustered and sensitivity of the 3D permutation method for oncogenes should be higher than that of TSGs, analysis of the mutation distribution can identify TSGs. The functional analysis of close mutations in the TSGs could help define new motifs and critical regions. Previously, Ryslik *et al.* proposed methods to and successfully identified mutation clusters in the 3D structure^7^,^8^. Their methods focused on small mutation clusters, which can be candidates for drug development, and majority of the identified genes were oncogenes. In this study, we examined the skew of mutation positions in the proteins, and identified both oncogenes and TSGs. We consider that the 3D permutation method we implement is more straight-forward and, as evident above, can identify TSGs.

In addition to identifying genes that showed a significant skew in mutation distributions, we were also able to identify skews in domains known to be important. For example, the RNase III domain of DICER1, which is a known mutational hotspot domain^37^, showed significant skew of mutation distribution (Fig. 3b). This suggests that mutation distributions are not uniform within a domain, and such analyses could contribute to the identification of functionally important amino acid positions within the domain.

We note that our method has several limitations to be assessed in future. First, this analysis is limited to mutations in genes with available 3D protein structures, and therefore genes/proteins without 3D information cannot be analyzed. It is also difficult to obtain 3D structures for the entire protein, for example, structures of transmembrane regions are difficult to examine. In our analysis, only 3,622 genes had 3 or more mutations in regions for which protein structures were available. Second, protein structures in PDB may not always accurately reflect protein structure *in vivo*. Third, errors in mutation call from sequencing analysis can affect these results, especially, if common germline SNVs are not called in multiple normal samples and identified in their matched cancer samples. The germline SNVs would be identified as clustered mutations. Since such types of errors should be rare, we excluded mutations found in the 1000 genomes common set and focused on genes that are mutated in ≥3% of the samples. However, a small number of false positives caused by germline SNVs may still be included in our list. Fourth, our method implicitly assumes that the mutation rate in a gene is uniform in the protein structure. This assumption should be appropriate for most genes, but may fail for some larger genes. For example, *TTN*, one of the largest genes, was significant in two cancers, and this may result from non-uniform mutation rate across the *TTN* gene region. Although analysis of the skew of distribution of synonymous mutations may make it possible to test the mutation rate variation in a gene, the number of the synonymous mutations in the downloaded data is too small to analyze. In the future, we will be able to obtain a larger number of mutations, and test the mutation rate variation with synonymous mutations. The contamination of germline SNVs and non-uniform mutation rates in a gene may explain the slight inflation seen in the Q-Q plots (Supplementary Fig. 2).

Together, our analysis systematically identified skewed mutation distributions in the 3D protein structure of 106 genes, which include many strong candidate driver genes with various biological functions. Further functional analysis of these candidates and the mutations should elucidate the mechanism of carcinogenesis and their status as driver genes. Since an accumulation of mutations in a protein can also indicate a good drug target, our methodology can be applied to future drug development, in addition to the identification of low frequency driver genes.

## Methods

### Data

Mutation data was downloaded from TCGA web site. MAF files with a data status of “No restrictions; all data available without limitations” were downloaded on December 19^th^, 2015 (Supplementary Table 1). Redundant SNVs in same sample were removed. To remove possible germline variations, we performed two kinds of SNVs filters: Filter 1, removal of somatic mutation candidates that were found in the 1000genome common SNV set, and Filter 2, removal of somatic mutation candidates that were found in dbSNP with a validation status of “YES”. The number of germline SNVs in dbSNP is much larger than these in 1000geome and filter 2 is the more conservative filter and, remove important hotspots in cancer as suggested in Jung *et al.*^39^. Indeed, known hotspots in *BRAS*, *KRAS* and *GNAS* were removed by filter 2. Therefore we discussed the results of filter 1 in the paper. All files in a cancer type were merged and annotated with gene information of GENCODE v19 (http://www.gencodegenes.org). Remaining SNVs that resulted in missense mutations were selected for analysis. Commands to visualize mutation position in the 3D structure in Chimera software were provided in the Supplementary Table 2.

Protein 3D structures were downloaded from PDB on August 7^th^, 2014.

Annotation of oncogenes and TSGs was based on Table S2A in Vogelstein *et al.*^10^. Cancer gene census was obtained from COSMIC.

### Comparison with other methods

We compared out methods with different methods; MutSigCV, MutSigCL, combined result of MutSig and OncodriveCLUST. MutSigCV is a gene burden test, and MutSigCL and OncodriveCLUST are methods to identify mutation clusters in the primary structure. The *P-values* of MutSigCV and MutSigCL were obtained from Lawrence *et al.*^22^. In the paper, 21 cancers were analyzed, and we selected four cancer types (BRCA, KIRC, LUSC, and UCEC), because mutation lists used in the study on the GRCh37 were available from TCGA database. *P-values* for MutSigCV, MutSigCL were obtained from Lawrence *et al.*^3^, and correction for the multiple testing was by Benjamini and Hochberg’s method^40^ with n = 18,388 according to Lawrence *et al.*^3^. We also compared the result of MutSigCL and OncodriveCLUST for LUSC and UCEC, whose results were available from previous publications^6^,^22^.

### Statistical methods

To quantify the skew of the mutation distribution, we applied a permutation test (Supplementary Fig. 1). Not all protein structures are human in origin, and most are not of the full length coding region. Because of this, we aligned the amino acid sequence of the protein structure to the translated reference genome using MAFFT^9^. Amino acid sequence aligned to human sequence with ratio of gaps to length of the alignment <0.5 and ratio of aligned sequence length to total sequence length ≥0.5 were used for the analysis. Using the alignment, we converted the positions of the mutations to those in the 3D structures.

Using the positions in the 3D proteins, we tested the skew in mutation position distribution. We calculated the pairwise Euclidian distances between mutations in the 3D protein structure, and averaged the distances (observed average distance). Given a gene with *n* mutations in the 3D structure, average pairwise Euclidian distances between *n* mutations was calculated as follows;


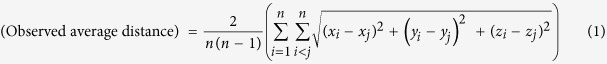


where (*x, y, z*) are location of amino acid residue in the 3D structure.

To test the significance of the observed average distance, we applied a permutation test. For gene with *n* mutations, *n* positions were randomly selected from the amino acid residues, and calculated average distances (simulated average distance) with the above formula. This step was repeated *m* times and the distribution of the simulated average distances was generated and used as the null distribution for the gene. (Fig. 1a, and Supplementary Fig. 1). We initially performed 10,000 permutations for each gene. To accurately estimate small *p-values*, genes with *p-value* ≤ 0.01 were reanalyzed using 1,000,000 permutations. If one gene had multiple 3D structures, the 3D structure with the lowest *p-value* was selected. Correction for multiple testing was done with the Benjamini and Hochberg’s method^40^.

We also analyzed the primary structure. We calculated the distance between mutations in the amino acid sequence. The observed distance was calculated as follows;


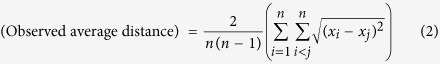


where *x* is position of the mutation in the primary structure. The calculation, selection of a *p-value* and correction of the multiple testing were carried out as done for the 3D permutation.

### Software

Program for the 3D permutation method is available (3D permutation; https://github.com/afujimoto/3Dpermutation.git).

### URLs and software

PDB; http://www.rcsb.org/pdb/home/home.do

Source of the 3D structures; ftp://ftp.wwpdb.org/pub/pdb/data/structures/divided/pdb/

Conversion of gene symbol to PDB id was based on ftp://ftp.ebi.ac.uk/pub/databases/uniprot/current_release/knowledgebase/complete/uniprot_sprot.dat.

MAFFT; http://mafft.cbrc.jp/alignment/software/

TCGA; http://cancergenome.nih.gov

COSMIC; http://cancer.sanger.ac.uk/cosmic

DAVID; http://david.abcc.ncifcrf.gov

Uniplot; http://www.uniprot.org

VMD; http://www.ks.uiuc.edu/Research/vmd/

Chimera; http://www.cgl.ucsf.edu/chimera/

REACTOME; http://www.reactome.org/

## Additional Information

**How to cite this article**: Fujimoto, A. *et al.* Systematic analysis of mutation distribution in three dimensional protein structures identifies cancer driver genes. *Sci. Rep.* **6**, 26483; doi: 10.1038/srep26483 (2016).

## Supplementary Material

Supplementary Information

Supplementary Dataset
