# Supplementary material for: Systematic analysis of mutation distribution in three dimensional protein structures identifies cancer driver genes
Source: Sci Rep. 2016 May 26;6:26483. doi: 10.1038/srep26483 (PMC4880911; doi:10.1038/srep26483)
Supplement: Supplementary Information [file srep26483-s1.doc]

Systematic analysis of mutation distribution in three dimensional protein structures identifies cancer driver genes

Akihiro Fujimoto, Yukinori Okada, Keith A. Boroevich, Tatsuhiko Tsunoda, Hiroaki Taniguchi, and Hidewaki Nakagawa

*Comparison between different filters for germline variants*

One of the most important initial steps of our analysis is removal of possible germline variants. If common germline SNVs are not called in multiple normal samples and identified in their matched cancer samples, the germline SNVs would be identified as clustered mutations. The clustered germline SNVs cause serious false positives issues.

To remove possible germline SNVs, we examined two independent filters: Filter 1, removal of somatic mutation candidates that were present in the 1000genome common SNVs set, and Filter 2, removal of somatic mutation candidates were found in dbSNP with a validation status of ”YES”. Since the number of germline SNVs in dbSNP is much larger than that of 1000genome, filter 2 is more conservative. The number of candidates (*q-value* < 0.1) was 188 (106 unique genes) for filter1, and 139 (89 unique genes) for filter2.

Filter2 removed some known hotspots, such as mutations in codon 600 of *BRAF*. Therefore, *BRAF* of THCA, and COAD were significant in filter1, but not significant in filter2. Similarly, *KRAS* and *NRAS* were only identified by filter1. Therefore, in this paper we mainly discuss the results of “filter1”. The difference in the results of the two filters is shown in **Supplementary Table 7.**

*Effect of minimum mutations count threshold selection*

To examined the influence of different minimum mutation count cutoff values, we compared the results when n=4, 5 and 6 with the results when n=3. An increase in the cutoff number decreased the number of genes analyzed, and influenced stringency of the correction of multiple testing. Results for n ≥ 4, 5 and 6 missed 25, 42, and 56 candidates, respectively, including *CTNNB1* in PRAD and *RB1* in GBM (**Supplementary Table2, 8**). On the other hand, genes with marginal *q-values*, including *NFE2L2* in BLCA (*q-value* = 0.15 in n≥3), *MAP2K1* in COAD,READ (*q-value* = 0.17 in n≥3) and *PIK3CA* in PRAD (*q-value* = 0.12 in n≥3), became significant (*q-value* < 0.1) (**Supplementary Table2, 8**). There are statistically marginal genes, and a change in cutoff threshold for mutations influences the results for these marginal genes (**Supplementary Table2, 8**).

*Adjustment of p-value with total number of 3D structures*

We applied the 3D permutation test to all 3D structures with ≥ 3 mutations. If multiple 3D structures existed for a single gene, the 3D structure with the lowest *p-value* was selected. The total number of tests in the entire analysis was equal to the number of all testable 3D structures. Therefore, we also performed multiple testing correction with the total number of the 3D structures, and the adjusted *p-values* were reported in the **Supplementary Table2**. One hundred twenty-one genes (60 unique genes) were significant (*q-value* < 0.1). We consider this the most stringent result in our method. However, each 3D structure of a gene was often similar, and the each *p-value* was not independent. Therefore, we discussed the result from the adjusted minimum *p-values* in the paper.

**Supplementary Figure 1. 3D permutation method procedure.** 3D protein structure was obtained from PDB. Since amino acid translation of the reference genome sequence is not always consistent with that of PDB, both amino acid sequences were aligned using MAFFT and mutation positions were converted (Mutations are shown in red. The mutation denoted by a “*” was not in the amino acid sequence in 3D structure and therefore was not analyzed). Observed average distance between mutations was calculated. The distance was evaluated with the permutation test. Mutations in *PTEN* in BRCA are shown as an example.

**Supplementary Figure 2. Q-Q plot of *p-values* from the 3D permutation test.** (a) ACC. (b) BLCA. (c) BRCA. (d) CESC. (e) COAD, READ. (f) GBM. (g) HNSC. (h) KICH. (i) KIRC. For genes with multiple 3D structures, 3D permutation analysis was done for all 3D structures and multiple *P-values* were obtained. We randomly selected one of these *P-values* for the Q-Q plots. (Please note that minimum *P-values* were selected for the other analyses).

**Supplementary Figure 2. Q-Q plot of *p-values* from the 3D permutation test.** (j) KIRP. (k) LIHC. (l) LUAD. (m) LUSC. (n) OV. (o) PRAD. (p) SKCM. (q) STAD. (r) THCA. For genes with multiple 3D structures, 3D permutation analysis was done for all 3D structures and multiple *P-values* were obtained. We randomly selected one of these *P-values* for the Q-Q plots. (Please note that minimum *P-values* were selected for the other analyses).

**Supplementary Figure 2. Q-Q plot of *p-values* from the 3D permutation test.** (s) UCEC. (t) UCS. Genes with multiple 3D structures, 3D permutation analysis was done for all 3D structures and multiple *P-values* were obtained. We randomly selected one these *P-values* for the Q-Q plots. (Please note that minimum *P-values* were selected for the other analyses).

**Supplementary Figure 3. Comparison with the other prediction methods.** (a) Comparison with a gene burden test. Comparison between the result of the MutSigCV and that of the 3D permutation methods for BRCA, KIRC, LUSC, and UCEC are shown.

**Supplementary Figure 3. Comparison with the other prediction methods.** (b) Comparison with other methods for identifying mutation clusters. Comparison among the MutSigCL and, the OncodriveCLUST and the 3D permutation method for LUSC, and UCEC are shown.

**Supplementary Figure 3. Comparison with the other prediction methods.** (c) Comparison with MutSig. Comparison between the result of the combined MutSig result and that of the 3D permutation methods for BRCA, KIRC, LUSC, and UCEC are shown.

**Supplementary Figure 4. Mutations in *CDKN2A* gene.** Mutations in the SKCM, HNSC, LUAD, and BLCA samples are shown in red, blue, orange and yellow, respectively. In HNSC, an additional hotspot was identified.

**Supplementary Figure 5. Comparison between 3D and 1D permutation.** (a) ACC (b) BLCA (c) BRCA (d) CESC (e) COAD, READ (f) GBM (g) HNSC (h) KICH (i) KIRC. Genes mutated in more than 3% of the samples are shown. Genes annotated as oncogenes and TSGs are shown by the triangles and diamonds, respectively. Please note that 1D permutation was done for entire coding regions, but 3D permutation analysis was limited to coding region in the available 3D protein structures. Significant genes by 1D permutation, 3D permutation, and both methods were shown in blue, pink and red, respectively.

**Supplementary Figure 5. Comparison between 3D and 1D permutation.** (j) KIRP (k) LIHC (l) LUAD (m) LUSC (n) OV (o) PRAD (p) SKCM (q) STAD (r) UCEC. Genes mutated in more than 3% of the samples are shown. Genes annotated as oncogenes and TSGs are shown by the triangles and diamonds, respectively. Please note that 1D permutation was done for entire coding regions, but 3D permutation analysis was limited to coding region in the available 3D protein structures. THCA did not have genes with mutations in > 3% of the samples, and therefore a figure was not generated. Significant genes by 1D permutation, 3D permutation, and both methods were shown in blue, pink and red, respectively.

**Supplementary Figure 5. Comparison between 3D and 1D permutation.** (s) UCS. Genes mutated in more than 3% of the samples are shown. Genes annotated as oncogenes and TSGs are shown by the triangles and diamonds, respectively. Please note that 1D permutation was done for entire coding regions, but 3D permutation analysis was limited to coding region in the available 3D protein structures. Significant genes by 1D permutation, 3D permutation, and both methods were shown in blue, pink and red, respectively.

**Supplementary Figure 6. Comparison of the analyses with primary and 3D structure.** 1D - genes identified only by 1D permutation test. 3D - genes identified only by 3D permutation test. Common - genes identified by the both tests. (a) Proportion of TSGs. Proportion of TSGs in the gene set identified by the both methods was significantly larger than that by 1D test (1D vs. Common; Fisher’s exact test *p-value*=0.023; odds ratio=8.5, and 3D vs. Common; not significant). (b) Proportion of oncogenes. Proportion of oncogenes in the gene set identified by both methods was significantly larger than that by 1D and 3D test (1D vs. Common; *p-value*=0.00066; odds ratio=16.1, and 3D vs. Common; *p-value*=0.00044; odds ratio=7.0). (c) Proportion of genes in COSMIC cancer genes. Proportion of COSMIC cancer genes was significantly different between 1D, 3D, and the common set. (1D vs. Common; *p-value*=5.7×10^-8^; odds ratio=45.1, 3D vs. Common; *p-value*=4.7×10^-5^; odds ratio=5.7, 1D vs. 3D; *p-value*=0.028; odds ratio=8.1). (d) Venn diagram of the result. Forty-seven genes were commonly identified by the both methods.

**Supplementary Figure 7. Examples of the significant genes identified by 3D permutation.** Amino acid sequence and 3D structure are shown. Recurrently mutated codons are indicated by square in the amino acid sequence, and the positions of the codon are shown in the 3D structures. (a) *ERBB2*. BLCA: *q-value*_3D_ = < 10^-6^, *q-value*_1D_ = n.s. (b) *KIT.* SKCM: *q-value*_3D_ = 0.0018, *q-value*_1D_ = n.s. (c) *HNF1A.* LIHC: *q-value*_3D_ = 0.048, *q-value*_1D_ = n.s. (d) *CUL1*. BLCA: *q-value*_3D_ = 0.035. *q-value*_1D_ = n.s.

**Supplementary Figure 7. Examples of the significant genes identified by 3D permutation.** (e) *GNAS*. SKCM: *q-value*_3D_ = 0.0018, *q-value*_1D_ = n.s.
